# Reduced menthol sensitivity in a prodromal Parkinson’s disease model induced by intranasal rotenone treatment

**DOI:** 10.3389/fncel.2024.1345651

**Published:** 2024-02-06

**Authors:** Hajime Sato, Keitaro Satoh, Kazunori Nozaki, Misato Yugawa, Takafumi Kato, Hiroki Toyoda, Ayano Katagiri, Naoto Suda, Kazunori Adachi

**Affiliations:** ^1^Division of Pharmacology, Meikai University School of Dentistry, Sakado, Japan; ^2^Division of Medical Information, Osaka University Dental Hospital, Suita, Japan; ^3^Division of Orthodontics, Meikai University School of Dentistry, Sakado, Japan; ^4^Department of Oral Physiology, Osaka University Graduate School of Dentistry, Suita, Japan

**Keywords:** Parkinson’s disease, rotenone, menthol, temperature sensitivity, seeking behavior, aversion

## Abstract

Parkinson’s disease (PD) is a neurodegenerative disorder characterized by motor symptoms, and it is associated with several prodromal non-motor symptoms, including an impaired sense of smell, taste and touch. We previously reported that bitter taste impairments occur independently of olfactory impairments in an early-stage PD animal model using short-term intranasal rotenone-treated mice. Cool temperatures also affect bitter taste perception, but it remains unclear whether or not bitter taste impairments result from an altered sensitivity for intraoral cool stimuli. We examined disturbances in the intraoral menthol sensitivity, such as coolness at low concentrations of menthol, using a brief-access test. Once a day, one solution from the 7-concentration series of (-)-menthol (0–2.3 mM) or the bitter taste quinine-HCl (0.3 mM) was randomly presented 20 times for 10 s to water-deprived mice before and 1 week after rotenone treatment. The total number of licks within 20 times was significantly decreased with the presentation of 2.3 mM menthol and quinine-HCl, compared to distilled water in untreated mice, but not in rotenone-treated mice. The correlation between the licks for quinine-HCl and that for menthol was increased after rotenone treatment. In contrast, the 2-bottle choice test for 48 h clarified that menthol sensitivity was increased after rotenone treatment. Furthermore, a thermal place preference test revealed that seeking behavior toward a cold-floored room was increased in the rotenone-treated mice despite the unchanged plantar cutaneous cold sensitivity. These results suggest that taste impairments in this model mice are at least partly due to intraoral somatosensory impairments, accompanied by peripheral/central malfunction.

## Introduction

Parkinson’s disease (PD) causes not only motor symptoms, but also prodromal non-motor symptoms, including sensory impairments (e.g., impaired senses of smell, taste, and/or touch). Olfactory impairments in PD patients are the most common prodromal symptoms, while taste impairments similarly occur in the early to middle stage of PD (Oppo et al., 2020; Melis et al., 2021). In addition, quantitative sensory testing in PD patients has revealed somatosensory impairments, for example, an increased cold detection threshold in the foot and hand (Nolano et al., 2008; Klatt-Schreiner et al., 2020). In our previous study, short-term intranasal rotenone-treated mice exhibited both olfactory and bitter taste impairments before motor deficits and neurodegeneration in the substantia nigra (SN) and the ventral tegmental area (VTA), implying a presumed animal model in the early-stage of PD (Yin et al., 2022). The bitter taste impairment in this PD model mice occurred independently of olfactory impairments, because bitter taste sensitivity itself in mice was not changed by olfaction (Inui-Yamamoto et al., 2020). However, it remains unclear whether this impairment is related to an altered intraoral temperature sensation.

Cool or cold temperatures impact bitter taste perception in human subjects (Green and Andrew, 2017), although the underlying mechanisms are not fully understood. The gustatory primary sensory nerve, the chorda tympani (CT) nerve, responds maximumly to quinine-HCl (QHCl, bitter taste) at 35–39°C, and responses are gradually decreased with a reduction in the intraoral temperature (Lu et al., 2016). In addition, bitter taste-responsive neurons in the nucleus tractus solitarius (NTS), the second-order gustatory system, are decreased in the number of spikes shown along with intraoral cooling (Lemon, 2017). Intraoral low temperatures are sensed through low-temperature sensors, such as the transient receptor potential (TRP) melastin 8 (TRPM8) and TRP ankyrin 1 (TRPA1) channel in the intraoral regions (Pan et al., 2018). Interestingly, menthol, a cooling agent in peppermints, which acts via the TRPM8 and TRPA1 channels, decreases the bitter taste perception of nicotine in C57BL/6 male mice, as shown using 2-bottle choice tests (Fan et al., 2016). Therefore, the bitter taste impairments observed in the short-term intranasal rotenone-treated mice in this study may be partly due to an altered sensitivity for intraoral cool stimuli.

The aim of this study was to clarify the relationship between bitter taste and intraoral cool sensitivity in an early-stage PD animal model. The findings of this study suggested the involvement of peripheral and/or central malfunctions in taste impairments with PD. This study may provide valuable insights for complicated taste impairments and help clarify our understanding of the neuropathology of non-motor symptoms in PD.

## Materials and methods

All the animal experiments conducted in this study were approved by the Animal Ethics Committees of Meikai University School of Dentistry and were conducted according to the guidelines issued thereby for the care and use of laboratory animals (project identification code: A2313/A2323/B2201).

### Intranasal rotenone treatment for mice

A total of 24 male C57BL/6J mice, 20–25 weeks of age (Sankyo Labo Service Corporation, Tokyo, Japan) were housed with a 12-h light/dark cycle at a constant room temperature (20–24°C) and given *ad libitum* access to food. We previously described the method employed for the intranasal administration of rotenone (Toyoda et al., 2020; Yin et al., 2022). Briefly, rotenone (Sigma-Aldrich, MO, USA) was first dissolved in 100% dimethyl sulfoxide (DMSO; Fujifilm Wako Pure Chemical Corporation, Osaka, Japan) to derive a stock solution at a concentration of 0.05 M. After the stock solution was diluted with polyethylene glycol (NACALAI TESQUE, Kyoto, Japan) just before the administration, rotenone (0.35 mg/kg) was delivered into the right side of the nose cavity in the light anesthetized mice with 3% isoflurane (Wako Pure Chemical Industries, Osaka, Japan) once a day for 1 week using a handy-type electronic dispenser (Icomes Lab Co., Ltd, Iwate, Japan).

### Brief-access test for preference of different water temperature and chemicals

**FIGURE 1:**
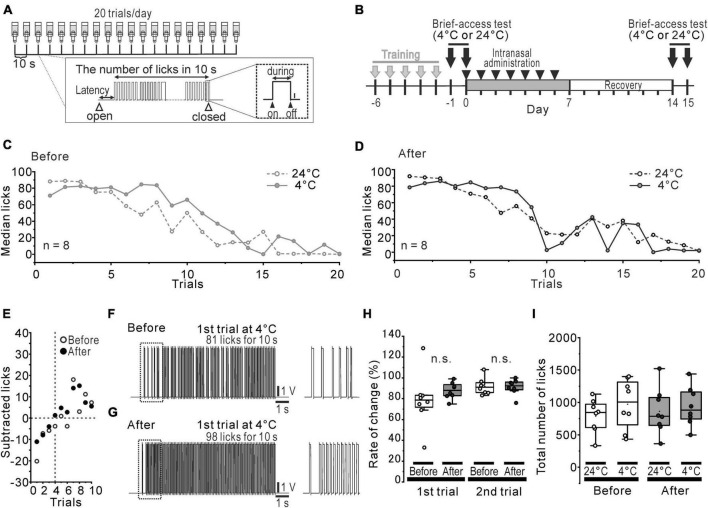
Slightly altered cold preference in water-deprived mice. **(A)** The protocol of the brief-access tests and the recorded waveform of licks in a trial. **(B)** The time course of the brief-access tests for 24°C and 4°C DW and the intranasal rotenone treatment. **(C, D)** Median licks in 24°C (open circles and dotted line) and 4°C (closed circles and solid line) DW presentation before **(C)** and after **(D)** the rotenone treatment. **(E)** The difference between the median number of licks for 4°C DW and that for 24°C DW. **(F, G)** The representative waveforms of licks in first trial of 4°C before **(F)** and after **(G)** the rotenone treatment. The dotted areas are enlarged on the right. **(H)** The percentage rate of change between the number of licks in 1st/2nd trials of 4°C and that of 24°C. **(I)** Total number of licks in 24°C and 4°C DW presentation before and after the rotenone treatment. DW, distilled water.

The brief-access test and training were conducted using a gustometer (LKT-1, MELQUEST, Toyama, Japan). Before the brief-access test, 18 h water-deprived mice were trained from 17:00 pm with room temperature distilled water (DW) for 5 days consecutively using the following protocol. Mice were allowed to access the spout for 10 s from the first lick, then the shutter was closed automatically. If a mouse did not lick the spout within 30 s, the shutter was closed once and reopened after 10 s. A total of 20 trials of 10 s presented in each training session were continuously run with 10 s inter-trial intervals (Figure 1A). After training, the solution was replaced with the test solution and the brief-access test was conducted using the same protocol before and 1 week after rotenone treatment (Figure 1B). On-, during- and off- signals, when the tongue was in contact with the spout, were recorded at a sampling frequency of 500 Hz through the A/D converter (PCD320A, KYOWA, Tokyo, Japan). The total number of licks and the latency to the first licks were examined. In order to conduct an assessment of the water temperature preference, 8 of the 18 h water-deprived mice were used. In order to avoid the influence of the previous experiment, cold (4°C) and room (24°C) temperature DW were randomly presented to the mice on different days before and after the rotenone treatment (Figure 1B). When estimating the sensitivity for 4°C DW during the initial trials, the ratio (%) was calculated by dividing the total number of licks for 4°C DW by that of 24°C DW for each trial. In another group (*n* = 8) of 18 h water-deprived mice, intraoral menthol and QHCl sensitivity was examined using 7-concentration series of (-)-menthol (0–2.3 mM, NACALAI TESQUE, Kyoto, Japan) and a consistent concentration of QHCl (0.3 mM, NACALAI TESQUE, Kyoto, Japan) on different days. In the test using menthol and QHCl, one solution from a series of 7 varying concentrations of (-)-menthol or 0.3 mM QHCl was randomly presented to the mice, once a day, and changing the concentration randomly each day, to the mice as a stimulus before and 1 week after the rotenone treatment. The cumulative lick curves were constructed across the sequential trials of menthol stimulus and standardized by dividing the cumulative number of licks on each trial by the total number of licks. In order to clearly address the relationship between bitter taste and menthol sensitivity, the coefficient of determination (*R*^2^) between the number of 0.3 mM QHCl licks at that of 1.5, 2.0, 2.3 mM menthol licks and the latency to the first licks in 1.5, 2.0, 2.3 mM menthol was calculated in the mice for each concentration before and after the rotenone treatment.

### 2-bottle choice test

An additional non-water-deprived mice (*n* = 8) were involved in only 2-bottle choice tests to evaluate the post-ingestive effects of menthol. Mice were presented with two bottles for 10 days (Figure 4). One bottle contained DW and the other contained any one of 4 concentrations of (-)-menthol [0.064 (10), 0.32 (50), 0.64 (100), 1.28 (200) mM (μg/mL)] (Fan et al., 2016). The menthol solutions were presented in a randomized order and the bottles were left in the cage calculated by dividing the consumed-volume of the DW or menthol solutions by the total consumed-volume of the DW and the menthol solutions.

### Thermal place preference test

The same group that went through the 2-bottle choice test also was presented with a thermal place preference test (TPPT) (Figure 4). The TPPT apparatus formed a single open topped enclosure (L 150 × W 150 × H 150 mm) and consisted of two rooms (150 mm × 150 mm each). The temperature resolution of the floor in these rooms was ± 0.1°C. One floor, in the reference room, was adjusted to the target floor temperature, whereas the other floor, the test room, was adjusted to the specific temperature of interest between 7 and 50°C. The mice were allowed to freely move from one to the other room through the hole in the partition between two rooms. For 2 days before TPPT, the mice were habituated in the apparatus for 5 min/day. On the day of the test, first, free moving for 10 min was examined under the setting at 25°C for both floors 4 h before TPPT. Next, TPPT was conducted under the setting at 10°C and 25°C for the test and reference floors, respectively. The mice were forcibly placed in the center of the test room 5 times, at 2 min intervals, and we recorded the free movements of the mice for 10 min using an infrared camera mounted above the apparatus. The transfer time from the test room to the reference room, the total stay time on the test floor, and the number of crossings through the hole from the test room to the reference room were measured for each animal by custom made software.

### Data expression and statistical analysis

All the data were expressed using box-whisker plots and considered statistically significant at *p* < 0.05. In general, data were statistically analyzed by two-way repeated-measures fractional ANOVA and two-way fractional ANOVA, followed by the Turkey honestly significant difference *post-hoc* test. The paired *t*-test was used for comparisons between 2 groups. Pearson’s correlation coefficient (r) and the coefficient of determination (*R*^2^) were used to assess the strength of the linear relationship between the total number of licks of QHCl and that/latency of menthol. Origin Pro 2023b software (LightStone Corp., MA, USA) was used for all of the statistical analyses.

## Results

### Preference for intraoral cold water

Orosensory-driven behavior for 4°C and 24°C DW was assessed using the brief-access tests (Figure 1B). In spite of the rotenone treatment, the median lick-trial curves across the trials showed the same pattern (Figures 1C, D) between before and after the rotenone treatment. When the median number of licks for 4°C DW was subtracted by that for 24°C DW, it indicated a negative value until the 3rd trial and then turned to a positive value up to the 10th trial for both before and after the rotenone treatment (Figure 1E). Although the inter-lick-interval of the 1st trials for 4°C DW slightly tended to narrow by the rotenone treatment (Figures 1F, G), the ratio (%) of the number of licks for 4°C DW to that of 24°C DW at the 1st and 2nd trial was not significantly changed by the rotenone treatment (Figure 1H). Moreover, there were no significant differences in the total number of licks between 4 and 24°C DW or between before and after the rotenone treatment (Figure 1I).

### Decreased avoidance behavior for menthol

**FIGURE 2:**
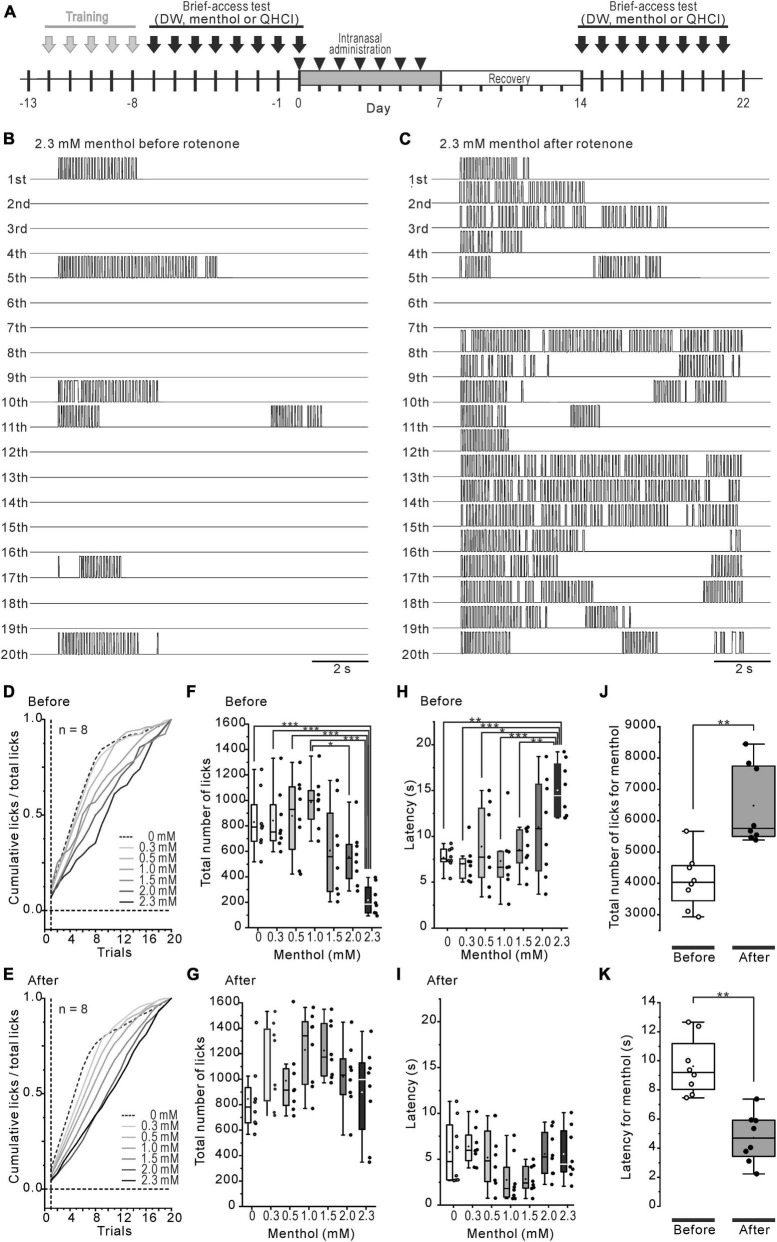
Decreased intraoral menthol sensitivity in rotenone-treated mice. **(A)** The time course of the brief-access tests for menthol and the intranasal rotenone treatment. **(B, C)** The representative waveforms of licks in all trials for 2.3 mM menthol before **(B)** and after **(C)** the rotenone treatment. **(D, E)** Mean standardized cumulative lick curves for the series of menthol concentrations before **(D)** and after **(E)** the rotenone treatment. **(F, G)** Total number of licks for the series of menthol concentrations before **(F)** and after **(G)** the rotenone treatment. **(H, I)** Latency of the 1st lick for the series of menthol concentrations before **(H)** and after **(I)** the rotenone treatment. **(J)** Total number of licks for menthol before and after the rotenone treatment. The paired *t*-test was used to compare the groups before and after the rotenone treatment for the total number of menthol licks. **(K)** Total latency of the 1st menthol lick before and after the rotenone treatment. The paired *t*-test was used to compare the groups before and after the rotenone treatment for the menthol latency. *p < 0.05, **p < 0.01, ***p < 0.001.

The intraoral aversion for the 7-step concentration series of (-)-menthol was examined before and 1 week after the rotenone treatment using the brief-access tests (Figure 2A). The number of licks across 20 trials was increased more after the rotenone treatment than it was before the treatment (Figures 2B, C). The mean standardized cumulative lick curve for menthol was not altered by the rotenone treatment, showing a dose-dependent slope for menthol (Figures 2D, E). While the total number of licks before the rotenone treatment was significantly (*p* < 0.001) decreased in 2.3 mM menthol, compared to 0–1.5 mM (Figure 2F), that of after the rotenone treatment was not significantly altered (Figure 2G). This resulted in a significant increase (*p* < 0.01) in the total number of licks after the rotenone treatment compared to before the treatment (Figure 2J). Additionally, the average latency of the first lick for 2.3 mM menthol was significantly (*p* < 0.05) increased before rotenone treatment compared to 0–1.5 mM menthol (Figure 2H), but not after the treatment (Figure 2I). This caused a significant decrease (*p* < 0.01) in the latency in menthol after the rotenone treatment compared to before the rotenone treatment (Figure 2K).

### The correlation between menthol and QHCl sensitivity

**FIGURE 3:**
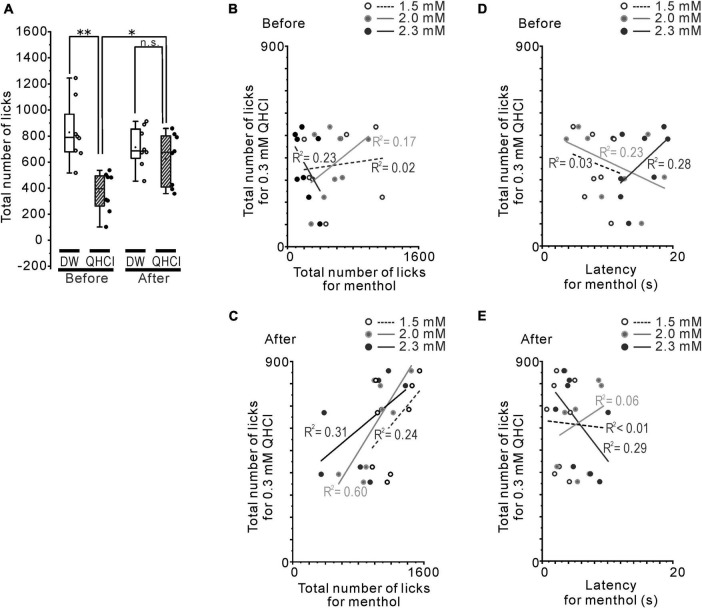
The relationship between QHCl and intraoral/intranasal menthol sensitivity. **(A)** The total number of QHCl licks at the 0.3 mM concentration or DW before and after the rotenone treatment. **(B)** The relationship between the number of QHCl licks at the 0.3 mM concentration and that for 1.5 (open circles), 2.0 (gray filled circles) or 2.3 (black filled circles) mM menthol in the mice before the rotenone treatment. **(C)** The relationship between the number of QHCl licks at the 0.3 mM concentration and that for 1.5 (open circles), 2.0 (gray filled circles) or 2.3 (black filled circles) mM menthol in the mice after the rotenone treatment. **(D)** The relationship between the number of QHCl licks at the 0.3 mM concentration and the latency for 1.5 (open circles), 2.0 (gray filled circles) or 2.3 (black filled circles) mM menthol in the mice before the rotenone treatment. **(E)** The relationship between the number of QHCl licks at the 0.3 mM concentration and the latency for 1.5 (open circles), 2.0 (gray filled circles) or 2.3 (black filled circles) mM menthol in the mice after the rotenone treatment. R square is the coefficient of determination. DW, distilled water. *p < 0.05, **p < 0.01.

Next, we confirmed that the altered intraoral menthol sensitivity was related to the bitter taste sensitivity. The avoidance behavior for 0.3 mM QHCl disappeared in the rotenone-treated mice (Figure 3A), indicating the reduction of bitter taste sensitivity in these mice. This result was also demonstrated in our previous study (Yin et al., 2022). The correlation between the total number of licks for 0.3 mM QHCl and that for 1.5, 2.0, and 2.3 mM menthol was positively increased after the rotenone treatment (*r* = 0.49, 0.77 and 0.55, *R*^2^ = 0.24, 0.60 and 0.31, respectively) compared to before the rotenone treatment (*r* = 0.12, 0.42, and −0.48, *R*^2^ = 0.02, 0.17 and 0.23, respectively) (Figures 3B, C). In contrast, the linear relationship between the total number of licks for 0.3 mM QHCl and the latency for 2.3 mM menthol shifted from positive (*r* = 0.53) to negative (*r* = −0.54) after the rotenone treatment, although there were no differences in the *R*^2^ between before (*R*^2^ = 0.28) and after (*R*^2^ = 0.29) the rotenone treatment (Figures 3D, E).

### The change in post-ingestive effects after menthol intake

**FIGURE 4:**
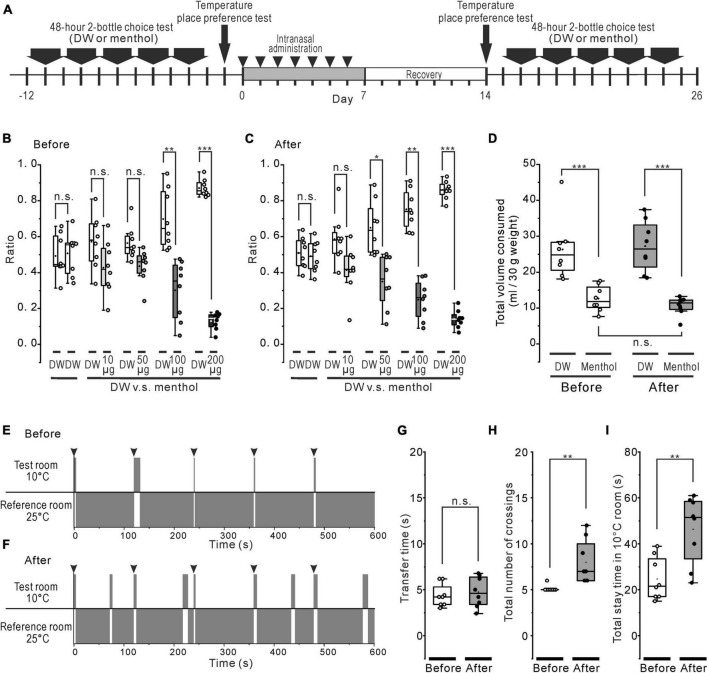
Systemic effect of the rotenone treatment on menthol or cold sensitivity. **(A)** The time course of 48-h 2-bottle choice tests, temperature (cold) place preference test, and the intranasal rotenone treatment. **(B, C)** Preference ratio for series of menthol concentration before **(B)** and after **(C)** the rotenone treatment. **(D)** Total consumed volumes for DW and all series of menthol concentration before and after the rotenone treatment. **(E, F)** The representative scheme of the cold place preference tests before **(E)** and after **(F)** the rotenone treatment. The mice were forcibly placed in the center of the test room 5 times, at 2 min intervals (arrowheads). **(G)** Transfer time from the 10°C floored room (test room) to the 25°C floored room (reference room) before and after the rotenone treatment. The paired t-test was used to compare the groups before and after the rotenone treatment for the transfer time. **(H)** The total number of crossings between the 10°C and the 25°C floored room before and after the rotenone treatment. The paired *t*-test was used to compare the groups before and after the rotenone treatment for the total number of crossings. **(I)** The total stay time in the 10°C floored room before and after the rotenone treatment. The paired *t*-test was used to compare the groups before and after the rotenone treatment for the total stay time. DW, distilled water. *p < 0.05, **p < 0.01, ***p < 0.001.

Before and after the rotenone treatment, the mice were given the choice between DW and a series of different concentrations of menthol solutions (Figure 4A). The untreated mice consumed significantly less (*p* < 0.01) menthol solutions at all concentrations of menthol exceeding 0.64 mM (100 μg/mL) (Figure 4B), while the rotenone-treated mice significantly avoided (*p* < 0.05) weakly aversive concentrations from 0.32 mM (50 μg/mL) of menthol (Figure 4C). However, there were no significant changes in the total consumed volume of menthol between before and after the rotenone treatment (Figure 4D).

### Intact avoidance, and increased seeking behavior, for cold stimuli

Thermal place preference test (TPPT) was conducted in the mice before and after the rotenone treatment (Figure 4A). When the untreated mice were forcibly placed on the center of the 10°C floor (test room), they showed a strong avoidance to the cold floor (Figure 4E), as moving quickly out (< about 5 s) to the 25°C floored room (reference room) and the mice showed a tendency to stay in that reference room (Supplementary Video 1). The rotenone-treated mice also avoided the cold-floored (test) room (Figure 4F) without changing the transfer time as well as the untreated mice (Figure 4G). However, the rotenone model mice spontaneously re-entered many times in the cold-floored room (Supplementary Video 2), resulting in a significant increase (*p* < 0.01) in the total number of crossings and the total stay time in the 10°C floored room (Figures 4H, I).

## Discussion

Assuming that the cold sensitivity in rotenone-treated mice was impaired, the reduction in the avoidance behavior for 4°C DW should be clearly observed, at least in initial trials of the brief-access test. However, the mice just showed a slight avoidance of cold water in the initial trials, but moderately preferred it during all of the trials, in spite of the rotenone treatment (Figure 1). The rotenone-treated mice exhibited a decreased intraoral sensitivity for menthol as well as bitter taste impairments (Figure 2), showing a positive correlation between intraoral menthol and bitter taste sensitivity (Figure 3). The disturbances for menthol odor were also slightly correlated with bitter taste sensitivity in the rotenone-treated mice. In contrast, the 2-bottle choice test revealed an increased post-ingestive effect of menthol on the rotenone-treated mice (Figure 4). Although avoidance behavior for the cold stimuli to the plantar regions using TPPT was not changed in the rotenone-treated mice, this aversion-seeking behavior was significantly increased (Figure 4). These results suggested that bitter taste impairments in rotenone-treated mice were caused by not only impaired taste sensitivity itself, but also impaired intraoral cool sensitivity, indirectly associated with olfactory impairments, gastrointestinal disturbances and the increased aversion-seeking behavior, derived from peripheral and/or central malfunction.

### Presumed involvement of the TRP channels in the disturbance of menthol sensitivity

Homozygous genetic deficiency for Trpa1 (TRPA1*^KO^*) mice, but not TRPM8*^KO^*, exhibit a reduced aversion to menthol in the brief-access tests (Lemon et al., 2019), suggesting the TRPA1 channel contributes to sensory-guided avoidance behavior for menthol in C57BL/6 mice. Consistent with a previous study (Lemon et al., 2019), the rotenone-treated mice exhibited an increased number of licks at high concentrations of menthol (Figure 2G), but with a different pattern of licks, compared with the TRPA1*^KO^* mice across trials (Lemon et al., 2019), as shown in the mean standardized cumulative lick curves for all menthol concentrations (Figure 2D). As shown in our previous study (Yin et al., 2022), the rotenone-treated mice in the present study exhibited a bitter taste impairment for QHCl. This bitter taste sensitivity was correlated with the menthol sensitivity in the rotenone-treated mice (Figure 3C), but not with the TRPA1*^KO^* mice (Lemon et al., 2019). Furthermore, the dose-consumption relationship for menthol in the 2-bottle choice tests was shifted to the lower concentrations of menthol in the rotenone-treated mice (Figure 4C). This was also seen in the TRPM8*^KO^* mice (Fan et al., 2016). Our results imply mild functional impairments in both of the TRPA1 and TRPM8 channels in the rotenone-treated mice.

Both of the TRPA1 and TRPM8 channels are activated by menthol, although whether or not they are implicated in sensing cool/cold temperature still remains an open question (Leijon et al., 2019). Some trigeminal ganglion (TG) neurons, which were activated by intraoral cooling, were not stimulated by menthol. The TRPA1 and TRPM8 channels are expressed not only in somatosensory neurons (e.g., TG neurons), but also in the membranes of mucosal cells (Moayedi et al., 2022). It is suggested that menthol directly stimulates epithelial cells and secondarily activates trigeminal sensory fibers. An intranasal administration of rotenone can directly affect all intraoral tissues and TG neurons through the trigeminal nerve pathway (Thorne et al., 2004; Johnson et al., 2010). Our results suggest that the expression in both the TRPA1 and TRPM8 channels is changed at the TG neurons, their axon terminals and/or mucosal cells of the intraoral regions. In contrast, the TRP channel also exhibited a widespread expression in the brain areas involved in the pathophysiology of PD (Vaidya and Sharma, 2020), but the changes in the central expression of the TRPA1 and TRPM8 channels still remains unknown in PD. Our study sheds some light on the importance of conducting future investigations to clarify the involvement of the TRPA1 and TRPM8 channels in the peripheral or central regions in regard to the complicated PD-related multi-sensory impairments.

### Presumed mechanisms underlying the impaired menthol and bitter taste sensitivity

Peripheral transection of the sensory branches of the trigeminal nerves in rats reduced the ingestive actions elicited by hedonic taste (1.0 and 0.03 mM sucrose), but left unchanged the aversive action elicited by aversive taste (0.03 and 0.3 mM QHCl) (Berridge and Fentress, 1985). Considering that rotenone-treated mice showed a reduced aversion for 0.3 mM QHCl (Figure 3A), taste-trigeminal interaction in peripheral and/or central gustatory pathway may be related to the reduction of both menthol and bitter taste sensitivity. Intraoral stimulation by a liquid solution excites both gustatory and somatosensory nerves [e.g., CT and the lingual nerve (LN), respectively] from the geniculate ganglion (GG) and TG, respectively. The gustatory information arrives at the rostral and intermitted NTS (rNTS and iNTS, respectively), while some LN directly also convey the somatosensory information at the rNTS (Felizardo et al., 2009). Considering the specific reduction of tyrosine hydroxylase immuno-reactive (TH-ir) neurons at both the rNTS and iNTS in our rotenone-treated mice (Yin et al., 2022), the local network in the NTS may be partly involved in the multi-sensory impairments in this model mice. In contrast, a small population of GG oral sensory neurons (10–20% PHOX2B positive neurons) are TH positive neurons, which have fibers that contact to Type II/III taste cells on the tongue and the soft palate and project into the rNTS (Tang and Pierchala, 2022). Interestingly, they do not express *Ret*, the mechanosensory neuron-associated gene, suggesting that they are gustatory and not somatosensory neurons. The finding that some GG neurons respond to both taste and cold temperature (Yokota and Bradley, 2016, 2017; Lemon, 2017), may imply the existence of a reciprocal interaction between gustatory and somatosensory information in GG. Although it remains unclear whether TH-ir neurons/fibers in the GG are intact in the rotenone-treated mice, both taste (bitter) and intraoral thermal (cool) impairments may be caused by the disruptions in the GG neural cells crosstalk (cell-to-cell communication) between TH-ir gustatory and non-TH-ir somatosensory neurons or glia cells in the GG.

The decreased menthol and bitter taste sensitivity may be also caused by an increase in palatability. Assuming that the increase in the palatability of menthol is due to the rotenone treatment, the rotenone-treated mice may complete the majority of their licks to the high concentration of menthol within the first quarter of the 20 brief-access trials, as well as that to DW. However, the number of licks shown by the rotenone-treated mice to 2.3 mM menthol in 1st trial showed a pattern about the same as that of non-treated mice (compare Figures 2B, C, 1st trial), and then this was gradually increased along with the increase in the trial number (Figure 2C, 2nd to 20th trial), resulting in a linear mean standardized cumulative lick curve for 2.3 mM menthol (compare the black dotted and solid lines in Figure 2E). In addition, after the rotenone treatment, the mice showed more avoidance of the menthol solutions in the 2-bottle tests than that shown before the rotenone treatment, suggesting a decreased long-term preference. Therefore, the increase in palatability might be remotely related to the decreased menthol and bitter taste sensitivity in rotenone-treated mice. In fact, the mesolimbic system is associated with the change in palatability for hedonic taste, but not for aversive taste (Shimura et al., 2002, 2005; Shinohara et al., 2009). Although it remains unclear whether there is a neurodegeneration in the mesolimbic dopamine pathway from the VTA to the nucleus accumbens and amygdala, the VTA neuron itself was intact in this model mouse (Yin et al., 2022). When conducting further studies using a prodromal PD animal model, we will attempt to prove the causal involvement of the mesolimbic dopamine system in the taste and somatosensory dysfunction of PD.

### Presumed mechanisms underlying the enhanced aversion-seeking behavior

Some studies have suggested that cold water is more rewarding and the preference for cold water is dependent on hydration status, that is, water deprivation time (Gold and Prowse, 1974; Torregrossa et al., 2012). In this study, 18 h water-deprived mice also slightly preferred cold (4°C) water in spite of the rotenone treatment (Figure 1). This result implies that the reward system in the rotenone-treated mice may be intact, at least, based on our previous result that the number of TH-ir cells in the VTA was normal with short-term rotenone treatment (Yin et al., 2022). Furthermore, the reward system in this model may be enhanced because TH-ir neurites in the insular cortex (IC) from the VTA or SN were significantly reduced (Yin et al., 2022). It has been reported that the activation of agranular IC and mesolimbic dopamine release are related to the frequencies of nose pokes to receive aversive stimulus (a brief air-puff) in C57BL/6J and/6N mice strain (Yawata et al., 2023). In addition, optogenetic activation of the IC also enhances the reward-related place preference indirectly by recruiting the dopaminergic TH-ir neurons in the VTA (Girven et al., 2021). These results suggest that the IC regulates the aversion-seeking behavior and the reward-related place preference. Considering the enhancement of the aversion-seeking behavior using TPPT in the rotenone-treated mice (Figure 4F), the decrease in the TH-ir neurites in the IC from the VTA might be partly involved in the aversion-seeking behavior and aversion itself for bitter taste or menthol.

Finally, the possibility still remains that the impairment of cool/cold sensitivity itself in the intranasal or orofacial regions of the rotenone-treated mice resulted in increased aversion-seeking behavior. We are planning a future study that will reveal the details of the somatosensory function in rotenone-treated mice, but the results of present study may offer an opportunity to focus our attention on the intraoral/extraoral somatosensory function in PD patients and remind us of the significance of an intraoral/extraoral thermal test, in addition to the classical taste test (e.g., the taste strip or the whole-mouth taste test) as part of the early diagnosis process for PD.

## Data availability statement

The original contributions presented in this study are included in the article/Supplementary material, further inquiries can be directed to the corresponding author.

## Ethics statement

The animal study was approved by the Animal Ethics Committees of Meikai University School of Dentistry (project identification code: A2313/A2323/B2201). The study was conducted in accordance with the local legislation and institutional requirements.

## Author contributions

HS: Conceptualization, Data curation, Funding acquisition, Investigation, Methodology, Project administration, Resources, Supervision, Validation, Visualization, Writing – original draft, Writing – review & editing. KS: Data curation, Formal analysis, Investigation, Methodology, Writing – review & editing, Visualization. KN: Data curation, Formal analysis, Investigation, Methodology, Software, Writing – review & editing. MY: Investigation, Methodology, Writing – review & editing, Visualization. TK: Conceptualization, Writing – review & editing. HT: Conceptualization, Writing – review & editing. AK: Conceptualization, Writing – review & editing. NS: Conceptualization, Writing – review & editing. KA: Conceptualization, Methodology, Project administration, Supervision, Writing – review & editing.

## Supplementary material

Supplementary Video 1 This movie shows the free movement for 10 min for a representative mouse before the rotenone treatment. At first, the mouse was forcibly placed in the center of the 10°C floored room (test room).

Supplementary Video 2 This movie shows the free movement for 10 min for a representative mouse 1 week after the rotenone treatment. At first, the mouse was forcibly placed in the center of the 10°C floored room (test room).
